# RNA sequencing analysis to capture the transcriptome landscape during skin ulceration syndrome progression in sea cucumber *Apostichopus japonicus*

**DOI:** 10.1186/s12864-016-2810-3

**Published:** 2016-06-14

**Authors:** Aifu Yang, Zunchun Zhou, Yongjia Pan, Jingwei Jiang, Ying Dong, Xiaoyan Guan, Hongjuan Sun, Shan Gao, Zhong Chen

**Affiliations:** Liaoning Key Lab of Marine Fishery Molecular Biology, Liaoning Ocean and Fisheries Science Research Institute, Dalian, Liaoning 116023 Peoples’ Republic of China

**Keywords:** Sea cucumber (*Apostichopus japonicus*), Skin ulceration syndrome, RNA sequencing, Dynamic expression profiles

## Abstract

**Background:**

Sea cucumber *Apostichopus japonicus* is an important economic species in China, which is affected by various diseases; skin ulceration syndrome (SUS) is the most serious. In this study, we characterized the transcriptomes in *A. japonicus* challenged with *Vibrio splendidus* to elucidate the changes in gene expression throughout the three stages of SUS progression.

**Results:**

RNA sequencing of 21 cDNA libraries from various tissues and developmental stages of SUS-affected *A. japonicus* yielded 553 million raw reads, of which 542 million high-quality reads were generated by deep-sequencing using the Illumina HiSeq™ 2000 platform. The reference transcriptome comprised a combination of the Illumina reads, 454 sequencing data and Sanger sequences obtained from the public database to generate 93,163 unigenes (average length, 1,052 bp; N50 = 1,575 bp); 33,860 were annotated. Transcriptome comparisons between healthy and SUS-affected *A. japonicus* revealed greater differences in gene expression profiles in the body walls (BW) than in the intestines (Int), respiratory trees (RT) and coelomocytes (C). Clustering of expression models revealed stable up-regulation as the main pattern occurring in the BW throughout the three stages of SUS progression. Significantly affected pathways were associated with signal transduction, immune system, cellular processes, development and metabolism. Ninety-two differentially expressed genes (DEGs) were divided into four functional categories: attachment/pathogen recognition (17), inflammatory reactions (38), oxidative stress response (7) and apoptosis (30). Using quantitative real-time PCR, twenty representative DEGs were selected to validate the sequencing results. The Pearson’s correlation coefficient (R) of the 20 DEGs ranged from 0.811 to 0.999, which confirmed the consistency and accuracy between these two approaches.

**Conclusions:**

Dynamic changes in global gene expression occur during SUS progression in *A. japonicus.* Elucidation of these changes is important in clarifying the molecular mechanisms associated with the development of SUS in sea cucumber.

**Electronic supplementary material:**

The online version of this article (doi:10.1186/s12864-016-2810-3) contains supplementary material, which is available to authorized users.

## Background

The transcriptome is a set of all RNA transcripts including rRNA, tRNA, mRNA and non-coding RNA produced in one type of cell or a population of certain types of cells at a particular stage in an organism [1]. Unlike the genome, which is roughly fixed for a certain cell type, the transcriptome is considered to be highly dynamic [2–7]. These transcriptomic changes are the prelude to the impact of protein translation on the phenotype of the organism. Transcriptome analysis is thus essential for elucidating the underlying molecular constituents of cells and tissues in various biological progresses. With the development of RNA-sequencing (RNA-seq) technology, it provides a far more precise measurement of the levels of transcripts and their isoforms than other methods [8]. Many biologically related issues, such as the expression levels of specific genes, the presence of novel transcripts and fusion transcripts and the events of differential splicing, allele-specific expression and RNA editing can be determined accurately by RNA-seq technology [1, 9–11]. Among several RNA-seq technologies, the Illumina sequencing platform is a particularly attractive approach to transcriptomic studies, because it is relatively low cost, and its coverage and depth are far greater than other currently available sequencing technologies [12–15]. Extensive studies have taken the advantage of the RNA-seq strategy to interpret the dynamic transcriptome in many species, such as maize [16–18], Drosophila [2], locust [4], Xenopus [5], and sea cucumber [6]. Other studies have also focused on whole transcriptome analysis to provide an improved understanding of the processes of tumorigenesis and cancer metastasis in human [19, 20].

The sea cucumber *A. japonicus* is one of the most valuable sea foods in East Asian countries because it has a high nutritional value. With increasing market demands for related products, sea cucumber aquaculture has developed rapidly in recent years, and has now become a vigorous industry along the northern coasts of China. However, various diseases in sea cucumber caused by bacteria, viruses or protozoa have severely limited the stable development of this important industry. Among these diseases, SUS is the most common and serious, usually resulting in high mortality. The SUS is characterized by skin ulceration, evisceration, general atrophy, swollen mouth and anorexia [15, 21, 22]. Some studies have focused mainly on the investigation, isolation and identification of the pathogens responsible for SUS epidemics [22–27]. Recently, investigations of the microRNAs (miRNAs) [15, 28] and genes involved in immune pathways [29] associated with SUS in *A. japonicus* have been reported. However, these molecular studies do not provide a comprehensive understanding of the immune response against SUS in *A. japonicus* and further studies of the molecular mechanisms involved in the SUS epidemics of *A. japonicus* are required.

SUS infection is a dynamic progress. After infection, skin ulceration begins with the appearance of one or more small white ulcerative specks, followed by deep and enlarged ulcerative lesions, leading to exposure of the underlying muscle and spicules. Finally, the ulcerative specks develop into extensive lesions and in severe case, many of the infected sea cucumbers lose the ability to attach to the tanks [21, 22, 26]. During the onset and progression of SUS, besides the appearance of obvious white ulceration on the skin, the molecular changes triggered by the disease comprise a process that is both dynamic and complicated. Systematic investigations of the multiple pathways involved in the multi-stage SUS progression and its polygenic regulation are required to clarify the origin and development of the disease. In the present study, we screened 21 cDNA libraries from populations of SUS-affected and healthy *A. japonicus* by high-throughput sequencing using the Illumina HiSeq™ 2000 platform. We compared the differentially expressed genes (DEGs) in four different tissue types (body walls, BW; intestines, Int; respiratory trees, RT; and coelomocytes, C) in SUS-affected and healthy samples, and also investigated the dynamic changes in the transcriptome of BW during the three-stage SUS progression. Furthermore, GO and KEGG enrichment analyses were conducted to elucidate the main functions of DEGs and to provide an overview of the regulation of genes during SUS progression. These results expand our understanding of the complex molecular mechanism of SUS, provide a framework for further investigations of SUS progression, and will be useful in determining strategies aimed at the prevention of SUS caused by bacterial pathogens in sea cucumber.

## Results

### Transcriptome sequencing and analysis

**Fig. 1:**
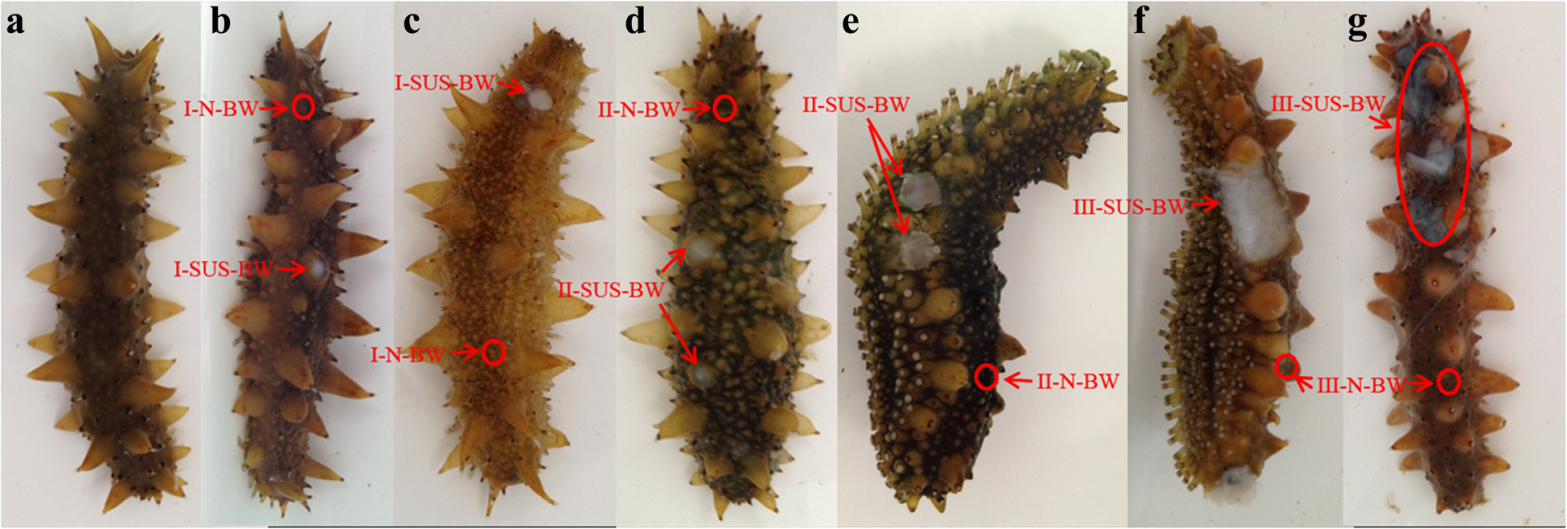
Features associated with each stage of SUS and indicators of the sampling positions. **a** Healthy *A. japonicus*. **b** and **c** SUS and its distal normal BW at stage I of SUS progression. **d** and **e** SUS and its distal normal BW at stage II of SUS progression. **f** and **g** SUS and its distal normal BW at stage III of SUS progression

To obtain a comprehensive gene expression profile for SUS progression in *A. japonicus,* we first constructed 13 cDNA libraries (from D1 to D13) for RNA-seq that represented the ulcerative body wall and distal normal tissue at all three SUS stages (Fig. 1), as well as four tissues (BW, Int, RT and C) obtained from SUS-affected and healthy individuals. Based on the analysis of the gene expression profiles in the first 13 cDNA libraries, we constructed eight cDNA libraries (from T1 to T8) for RNA-seq that represented the ulcerative BW of the three SUS stages and the BW of healthy individuals using the Illumina HiSeq™ 2000 platform. A total of 553 million (553,212,906) raw reads were generated from 21 libraries; these were deposited in the NCBI SRA database (accession number: SRP050068). After trimming the raw reads, a total of 542 million (541,944,042) high-quality clean reads were obtained (Table 1). The map reference transcriptome containing 161,174,656 Illumina reads of the first RNA-seq (Table 1), 104,067,712 Illumina reads from our previous studies [30], 1,076,411 pre-processed 454 sequences [31] and 4,786 expressed sequence tags (ESTs) from the public database were included in the *A. japonicus* transcriptome analysis (Table 2). According to the method described by Zhou et al., the Illumina reads were combined with the 454 sequencing data and Sanger sequences to improve the quality of the assembled transcriptome [30]. Assembly of these sequences generated 93,163 unigenes with an average length of 1,052 bp (N50 = 1,575 bp). The total assembled unigenes included 39,744 transcripts attributed to the different sequence splicing of 17,252 genes. Each of these unigenes contained at least two sequences with pairwise sequence similarity greater than 70 %. Of all the unigenes obtained, 30,315 showed significant matches to Nr, 11,771 to Nt databases, 26,412 to SwissProt, 21,316 to KEGG and 13,976 to GO. Altogether, 33,860 unigenes had significant matches, with at least one match to these databases for each of the unigenes (Table 2). Approximately 74.19 % to 78.84 % (Table 1) of the total clean reads from all the libraries were mapped to the 93,163 unigenes obtained from the reference transcriptome data.

**Table 1:** Summary of the alignment statistics for cDNA libraries in *A. japonicus*

Sample ID	Libraries name	Total Reads	Total Clean Reads (Percentage)	Total Mapped Reads (Percentage)
1	H-BW (D1)	12,378,414	12,098,662 (97.74 %)	9,410,905 (77.78 %)
2	H-Int (D2)	12,602,105	12,430,716 (98.64 %)	9,576,807 (77.04 %)
3	H-RT (D3)	12,674,851	12,498,671 (98.61 %)	9,454,890 (75.65 %)
4	H-C (D4)	13,579,708	12,972,695 (95.53 %)	9,914,340 (76.42 %)
5	I-SUS-BW (D5)	12,791,397	12,451,146 (97.34 %)	9,473,854 (76.09 %)
6	I-N-BW (D6)	11,709,219	11,400,096 (97.36 %)	8,611,908 (75.54 %)
7	II-SUS-BW (D7)	12,426,665	12,262,633 (98.68 %)	9,408,950 (76.73 %)
8	II-N-BW (D8)	11,529,198	11,225,980 (97.37 %)	8,409,997 (74.92 %)
9	II-SUS-Int (D9)	13,183,442	13,008,102 (98.67 %)	10,255,121 (78.84 %)
10	II-SUS-RT (D10)	13,080,922	12,887,324 (98.52 %)	10,057,462 (78.04 %)
11	II-SUS-C (D11)	13,220,635	13,044,801 (98.67 %)	10,064,650 (77.15 %)
12	III-SUS-BW (D12)	12,239,111	12,073,883 (98.65 %)	9,327,113 (77.25 %)
13	III-N-BW (D13)	13,050,949	12,819,947 (98.23 %)	9,831,997 (76.69 %)
Total reads for the first RNA-seq		164,466,616	161,174,656 (98.00 %)	123,797,994 (76.81 %)
14	H-BW (T1)	51,200,576	50,022,670 (97.7 %)	38,946,697 (77.86 %)
15	H-BW (T2)	44,371,450	43,357,830 (97.72 %)	32,165,535 (74.19 %)
16	I-SUS-BW (T3)	51,818,216	50,866,582 (98.16 %)	39,225,086 (77.11 %)
17	I-SUS-BW (T4)	49,027,132	47,952,352 (97.81 %)	37,436,180 (78.07 %)
18	II-SUS-BW (T5)	40,931,866	40,071,484 (97.9 %)	30,984,132 (77.32 %)
19	II-SUS-BW (T6)	55,174,172	53,899,574 (97.69 %)	42,222,134 (78.33 %)
20	III-SUS-BW (T7)	43,123,468	42,213,860 (97.89 %)	32,138,856 (76.13 %)
21	III-SUS-BW (T8)	53,099,410	52,385,034 (98.65 %)	40,650,674 (77.59 %)
Total reads for all RNA-seq		553,212,906	541,944,042 (97.96 %)	417,567,288 (77.05 %)

Note: *H* Healty sea cucumber, *BW* Body wall, *Int* Intestine, *RT* Respiratory tree, *C* Coelomocyte, *SUS* Skin ulceration syndrome, *I* SUS stage I, *II* SUS stage II, *III* SUS stage III, *N* Normal tissue

**Table 2:** Summary statistics of transcriptome assembly for *A. japonicus*.

Category	Count
Total Clean Reads	
Illumina reads (First RNA-seq)	161,174,656
Illumina reads [30]	104,067,712
454 reads	1,076,411
Sanger reads	4,786
Assembly Results	
No.of unigenes	93,163
Mean Length	1,052
N50	1,575
Annotation	
Nr database	30,315
Nt database	11,771
Swiss-Prot	26,412
KEGG	21,316
GO	13,976
ALL	33,860

### Analysis of the gene expression profiles during SUS progression

One of the primary goals of RNA-seq is to compare the gene expression levels from different samples. Analysis of the first 13 cDNA libraries revealed the sequential large-scale gene expression profiles of healthy and SUS-affected *A. japonicus* individuals, as well as the DEGs of ulcerative and normal BW in the same individuals. Transcripts with at least two-fold differences (log_2_Ratio ≥1, FDR ≤0.001) were classified as DEGs. As shown in Table 3, the largest number of DEGs was observed when the profiles of the Int, RT and C were compared to those of the BW in the healthy group (16,936, 17,164 and 16,692 DEGs, respectively), and the number of up-regulated DEGs was obviously higher than that of down-regulated DEGs*.* Interestingly, SUS caused a significant reduction in the number of DEGs between the BW and the other three tested tissues (Int, 8,647; RT, 6,888; and C, 6,072), with the number of down-regulated DEGs being greater than or similar to the number of up-regulated DEGs. It is noteworthy that the numbers of DEGs in the BW were higher in SUS-affected samples compared to those in healthy samples (I-SUS-BW vs. H-BW 9,854, II-SUS-BW vs. H-BW 10,182, III-SUS-BW vs. H-BW 9,726 and I-N-BW vs. H-BW 9,607, II-N-BW vs. H-BW 9,670, III-N-BW vs. H-BW 9,570 DEGs), but lower when SUS-affected samples obtained at the three infection stages were compared to each other (619, 514, and 946 in the SUS groups and 732, 499, and 961 in the normal groups). When the stage II SUS samples were compared to healthy samples, small numbers of DEGs were found in the Int (1,718), RT (3,279) and C (1,847), while more were found in the BW (II-SUS-BW vs. H-BW 10,182 and II-N-BW vs. H-BW 9,670). In addition, very few DEGs were found in the comparison of SUS and normal BW during the three SUS stages (646, 127 and 568 DEGs, respectively), and the lowest number of DEGs was screened at SUS stage II. These data demonstrated the following: (1) the differences in gene expression profiles between SUS-affected and healthy samples were larger in the BW than those in the Int, RT and C; (2) considerable differences in gene expression profiles were detected in the BW of SUS-affected individuals (including SUS stages I, II and III) compared to healthy individuals, while smaller differences in gene expression profiles were observed among the three stages of SUS progression; and (3) the number of DEGs between SUS and normal BW from the same individual exhibited minimum values at SUS stage II.

**Table 3:** The list of two-class DEGs among the first 13 libraries

Different tissues		Different SUS stages				SUS tissues vs normal tissues	
Libraries name	No.of DEGs	Libraries name	No.of DEGs	Libraries name	No.of DEGs	Libraries name	No.of DEGs
H-Int (D2) vs H-BW(D1)	↑12,639 ↓ 4,297 T 16,936	I-SUS-BW (D5) vs H-BW(D1)	↑7,401 ↓2,453 T 9,854	II-SUS-BW(D7) vs I-SUS-BW (D5)	↑340 ↓279 T 619	I-SUS-BW (D5) vs I-N-BW (D6)	↑442 ↓204 T 646
H-RT(D3) vs H-BW(D1)	↑ 13,923 ↓ 3,241 T 17,164	II-SUS-BW(D7) vs H-BW(D1)	↑7,705 ↓2,477 T 10,182	III-SUS-BW(D12) Vs II-SUS-BW(D7)	↑299 ↓215 T 514	II-SUS-BW (D7) vs II-N-BW (D8)	↑ 20 ↓107 T 127
H-C(D4) vs H-BW(D1)	↑ 12,294 ↓ 4,398 T 16,692	III-SUS-BW(D12) vs H-BW(D1)	↑7,229 ↓2,497 T 9,726	III-SUS-BW(D12) vs I-SUS-BW (D5)	↑493 ↓453 T 946	III-SUS-BW(D12) vs III-N-BW (D13)	↑307 ↓261 T 568
II-SUS-Int(D9) vs II-SUS-BW(D7)	↑3,758 ↓4,889 T 8,647	I-N-BW(D6) vs H-BW(D1)	↑7,805 ↓1,802 T 9,607	II-N-BW(D8) vs I-N-BW(D6)	↑437 ↓295 T 732	II-SUS-Int (D9) vs H-Int (D2)	↑ 581 ↓1,137 T 1,718
II-SUS-RT(D10) vs II-SUS-BW(D7)	↑3,523 ↓3,365 T 6,888	II-N-BW(D8) vs H-BW(D1)	↑7,662 ↓2,008 T 9,670	III-N-BW(D13) vs II-N-BW(D8)	↑205 ↓294 T 499	II-SUS-RT (D10) vs H-RT (D3)	↑ 871 ↓2,408 T 3,279
II-SUS-C(D11) vs II-SUS-BW(D7)	↑1,711 ↓4,361 T 6,072	III-N-BW(D13) vs H-BW(D1)	↑7,789 ↓1,781 T 9,570	III-N-BW(D13) vs I-N-BW(D6)	↑531 ↓430 T 961	II-SUS-C (D11) vs H-C (D4)	↑ 700 ↓1,147 T 1,847

### Stage-series gene expression modeling during SUS progression

**Fig. 2:**
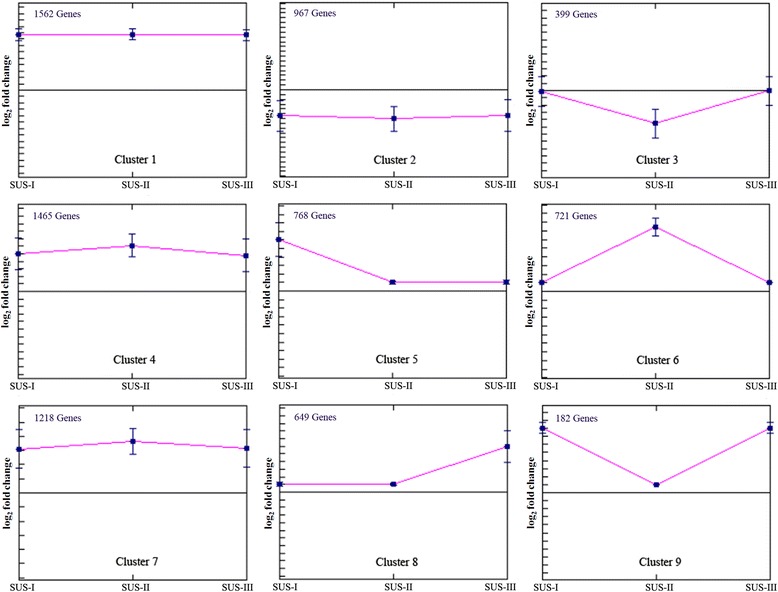
Dynamic expression patterns in *A. japonicus* during SUS progression. The expression profiles of the DEGs [the log_2_Ratio ≥1 and the RPKM >2 at a minimum of one time-point] were determined over the three stages of SUS progression by clustering analysis based on the K-means method using the Euclidean distance algorithm. The three points along the x-axis represent I-SUS-BW/H-BW, II-SUS-BW/H-BW and III-SUS-BW/H-BW. Each tick on the y-axis represents a value of 1. Midline represents 0. “+1” represents up-regulated expression. “-1” represents down-regulated expression

The patterns of DEGs for SUS stages I, II and III were assigned as three serial points in the progression of SUS. To classify the dynamics of the SUS development transcriptome on a global scale, we performed gene expression profile clustering. The seven representative expression patterns of SUS progression are presented in Fig. 2. Visual inspection of these expression models suggested the existence of a diverse and complex pattern of regulation during SUS progression. The first model that represented the main expression pattern comprised Cluster 1, Cluster 4 and Cluster 7, containing 4,245 DEGs. These clusters showed higher levels of DEGs compared to healthy samples and were sustained stably with advancing SUS progression. In contrast, Cluster 2 showed sustained lower levels of DEG expression compared to healthy samples at the three SUS stages. Three expression models based on Cluster 3, Cluster 6, and Cluster 9, revealed a similar expression pattern at SUS stages I and III, while a distinct expression pattern was observed during the transition period at SUS stage II. Moreover, Cluster 5, Cluster 6 and Cluster 8 presented significantly higher levels of expression specific for stages I, II and III, respectively.

**Fig. 3:**
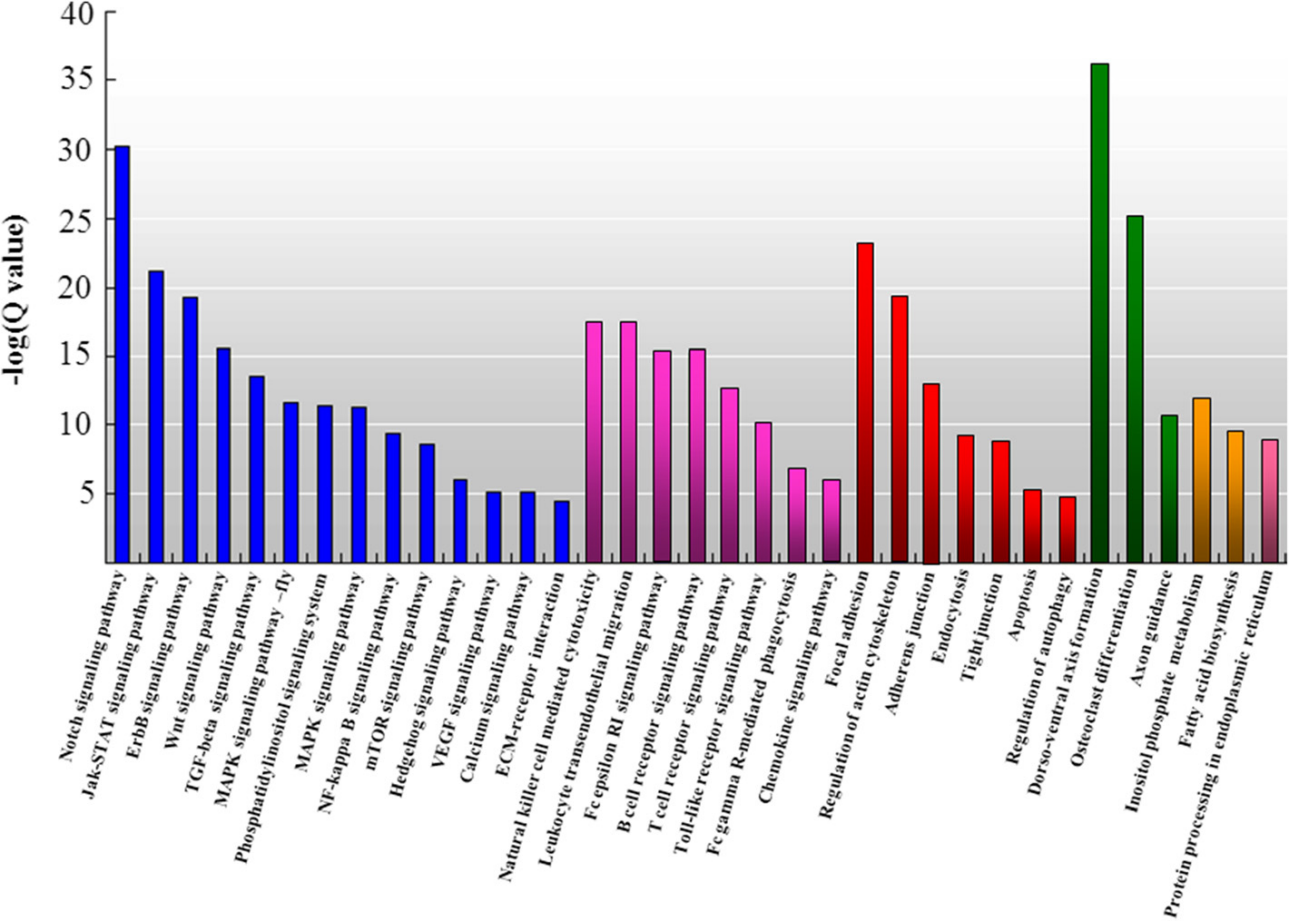
Significantly enriched pathway among the DEGs revealed by the up-regulated expression models in *A. japonicus* during SUS progression. Blue: Pathways related to signal transduction. Magenta: Pathways related to the immune system. Red: Pathways related to cellular processes. Green: Pathways related to development. Orange: Pathways related to metabolism. Pink: Pathways related to genetic information processing

GO and KEGG enrichment analysis of the stably up-regulated expression pattern (4,245 DEGs) during SUS progression showed 175 GO terms (25 GO terms in the category “cellular component”, 13 GO terms in the category “molecular function”, and 137 GO terms in the category “biological process”) (Additional file 1). GO analysis demonstrated that these DEGs with positive regulation function were involved in a broad range of physiological processes. The most notably abundant GO terms in the stably up-regulated expression model were “protein binding”, “signal transduction”, “cell communication”, “signaling” and “single organism signaling”. This main stage-series gene expression model of SUS progression associated with biological pathways was evaluated by enrichment analysis of DEGs. Significantly enriched pathways were identified and are presented in Fig. 3 (Additional files 2, 3, 4, 5 and 6; Q value ≤ 0.05). For the 35 significantly enriched pathways identified in this expression model, 14 were related to signal transduction. Eight significantly enriched pathways consisting of “natural killer cell mediated cytotoxicity”, “leukocyte transendothelial migration”, “Fc epsilon RI signaling pathway”, “B cell receptor signaling pathway”, “T cell receptor signaling pathway”, “Toll-like receptor signaling pathway (TLR pathway)”, “Fc gamma R-mediated phagocytosis” and “chemokine signaling pathway” were related to the immune system. Seven significantly enriched pathways were related to cellular processes, with apoptosis and regulation of autophagy pathways being noteworthy. Three significantly enriched pathways, “dorso-ventral axis formation”, “osteoclast differentiation” and “axon guidance”, were related to development.

### Investigation of DEGs during SUS progression

Based on the gene expression profiles in the first 13 cDNA libraries, we focused on the changes in the transcriptome in the BW of sea cucumber during the SUS progression from stage I to stage III. Thereafter, we re-sequenced samples from ulcerative BW of the three SUS stages and healthy BW. Compared to healthy *A. japonicus*, the number of DEGs in the BW of SUS stages I, II and III were 5,478, 5,589 and 4,437, respectively (Additional files 7 and 8). Combined with the results of the first 13 libraries, we further investigated the DEGs involved in the immune responses from extracellular interaction with bacteria to the activities within the nucleus. Based on GO and KEGG pathway annotations, manual blast and literature searches, 92 DEGs with Nr annotations were selected and divided into four main functional categories: (1) attachment/pathogen recognition (17 genes); (2) inflammatory reactions (38 genes); (3) oxidative stress response (7 genes); and (4) apoptosis (30 genes). A subset of these candidates is listed in Table 4. Among them, the vast majority of these DEGs were up-regulated in the process of SUS progression in *A. japonicus.* The comparison resulted in a trend concordance between the two sets of sequencing data.

**Table 4:** Special types of DEGs during SUS progression in *A. japonicus*

Gene name	First sequencing			Re-sequencing		
	SUS-I	SUS-II	SUS-III	SUS-I	SUS-II	SUS-III
Attachment/Pathogen recognition						
CLECT	−1.56	−1.79	−2.99	−3.27	−3.34	−3.77
CLECT isoform 3	−1.69	−2.01	−2.21	−3.27	−3.23	−3.64
CLEC19A	−1.71	−1.89	−2.12	−2.51	−2.68	−2.78
GalNAc-specific lectin	−1.21	−1.52	−1.91	−5.37	−6.05	−6.58
lactose-binding lectin l-2-like	^a^	^a^	−1.29	^a^	−2.96	−3.24
Fibrinogen-like protein A	−1.32	−1.25	−1.18	−2.74	−2.79	−2.18
SRCR protein	3.47	3.62	3.91	5.32	5.27	5.68
FNDC3A-like	7.49	7.51	6.39	5.09	5.08	4.91
Annexin A13	1.56	1.64	1.27	2.21	1.69	1.33
Mucin-2	6.21	5.49	7.66	8.78	8.38	8.64
Integrin alpha-1	2.14	1.85	1.8	3.80	3.20	3.23
Integrin alpha-9-like	4.6	4.02	5.08	5.45	4.77	4.65
Integrin Alpha-Lv1	5.88	6.23	7.08	6.23	5.31	5.5
Integrin beta-2	2.02	1.45	1.51	2.24	1.94	1.74
Integrin beta-C subunit	13.24	12.8	11.31	5.31	5.31	5.24
Integrin beta G subunit precursor	4.44	4.11	4.24	4.35	3.98	3.83
Tenascin-R-like	−1.67	−2.28	−2.04	−1.18	−1.77	−1.89
Inflammation Reactions						
Complement C3	2.82	2.93	3.15	3.68	3.55	3.39
Complement component 3-2	6.63	6.42	6.5	3.49	3.15	3.29
Complement Bf	1.29	1.63	1.64	3.3	3.45	3.41
Complement receptor type 2 isoform X1	2.71	3.43	2.87	3.42	4.25	3.51
IgGFc-binding protein	3.21	3.31	2.51	4.99	4.85	4.2
Collagen alpha-1 (XI) chain-like	5.12	5.27	5.11	5.99	5.91	5.81
Collagen alpha-2	7.11	6.34	6.03	5.93	5.06	5.13
Collagen alpha-5	4.51	3.62	3.31	5.02	4.52	4.06
MyD88	1.36	1.38	1.54	1.60	1.64	1.75
TRAF 1	2.29	2.63	2.69	4.02	4.1	4.42
TRAF5-like	5.46	7.65	5.94	2.44	2.93	2.57
IRAK4-like	3.65	3.57	3.91	3.7	3.09	2.86
NF-kB transcription factor Rel	3.25	3.78	3.48	3.64	4.11	3.91
NF-kB p105 subunit	3.45	3.54	3.82	4.17	4.22	4.4
MAPKKK	2.25	2.25	2.17	2.38	2.81	2.61
Serine/threonine-protein kinase TBK1-like	4.82	5.07	4.79	5.46	5.31	5.12
Stress-activated protein kinase JNK-like	4.24	4.21	4.33	3.89	3.63	4.02
TGF beta-activated kinase	2.89	2.98	2.96	4.35	4.21	4.53
TOLLIP	3.41	3.79	3.56	3.43	4.25	3.91
Zonadhesin-like	6.67	5.5	6.97	11.89	11.85	11.38
IFI27-like protein 2	−2.64	−2.47	−2.38	−3.02	−2.93	−3.17
Apolipoprotein B-100-like	3.47	3.83	4.22	2.93	3.18	3.56
Macrophage mannose receptor 1-like	3.01	3.25	3.18	4.3	4.63	4.72
Matrix metalloproteinase-9	5.72	4.84	5.42	8.41	7.71	8.15
Matrix metalloproteinase-24	3.91	3.71	4.01	2.41	1.41	1.57
Matrix metalloproteinase 24 preproprotein-like	4.52	3.43	3.59	3.91	3.71	4.01
Prostaglandin E synthase 2-like	1.26	1.42	1.23	1.51	1.77	1.85
Suppressor of cytokine signaling 2-like	5.33	4.89	5.18	4.95	4.45	4.81
Tumor protein p53-inducible protein 11-like	2.34	2.59	2.26	4.23	4.79	4.42
NFIL3	2.07	2.96	2.38	3.21	2.38	1.41
TMPRSS 5-like	10.34	10.37	10.79	10.39	10.41	10.83
LENG8 homolog	4.28	4.44	3.99	5.37	5.48	5.04
IGDCC4-like	3.76	3.08	3.15	1.87	1.95	1.88
LIG-3 isoform 2	2.22	2.51	2.41	3.45	3.94	3.63
Variable lymphocyte receptor	2.02	2.27	2.28	9.74	11.04	11.26
Thrombospondin-2	4.31	4.22	3.88	3.13	3.01	2.72
Thrombospondin-3	5.28	5.08	5.31	4.77	4.49	4.86
Major yolk protein	−2.63	−2.51	−2.13	−3.07	−3.03	−3.45
Oxidative Stress Response						
HSP 70	3.84	5.24	5.09	3.76	5.17	5.06
HSP 26	3.59	5.84	5.58	2.63	4.55	4.54
HSP 110	2.08	1.63	1.73	3.33	2.96	3.26
HSP 90	7.13	7.42	7.83	2.31	1.59	1.75
Dual oxidase 1	4.36	4.27	4.41	4.82	4.54	4.65
GPX1-like	2.09	1.92	1.62	2.72	2.50	2.65
Thioredoxin	2.33	2.19	2.32	2.91	2.7	3.38
Apoptosis/Autophagy/Lysosome/Phagosome						
Apoptosis regulator BAX-like	2.02	2.27	2.45	7.72	7.52	8.36
Apoptosis-inducing factor 2-like	2.08	2.14	1.99	2.21	2.48	2.01
Calpain-5	2.65	2.64	2.08	4.77	4.79	4.20
Calpain-7	1.96	1.94	1.99	3.06	3.12	3.08
Caspase-6-like	3.82	4.12	4.36	2.12	3.31	2.44
Cyclophilin B	1.97	1.96	1.91	1.90	2.03	2.30
Cytochrome c-like	−1.14	−1.32	−1.22	−1.65	−1.84	−1.75
Cytochrome c oxidase subunit 7C	−2.06	−1.95	−1.95	−2.57	−2.65	−2.76
BFAR -like	2.57	3.19	2.73	8.03	8.15	7.95
Bcl-2 protein	5.29	4.83	4.67	4.08	3.65	3.43
Bcl-2-like protein 1-like	2.25	2.58	2.32	4.04	4.34	4.14
BIRC2	3.28	2.9	3.01	3.8	3.52	3.42
BIRC 6-like	3.18	3.17	2.96	5.06	5.11	4.89
PDCD6IP -like	2.17	2.17	2.22	3.61	3.77	3.69
PDCD10 -like	3.79	3.47	3.54	4.41	4.29	4.32
DRAM 2-like	2.38	2.54	2.95	3.93	4.24	4.19
PDRG 1	−2.83	−2.83	−1.83	−3.81	−3.51	−3.13
Exportin-1-like	1.59	1.52	1.59	11.77	10.38	11.82
Exportin-T	1.37	1.49	1.61	1.64	1.42	1.59
Cathepsin D	7.13	7.47	7.44	6.13	7.16	8.09
Cathepsin L	1.72	1.83	1.79	3.18	3.84	4.71
Cathepsin B-like protease	3.08	3.19	3.25	3.23	3.37	3.57
Lysozyme-like	−1.51	^a^	−1.69	−3.38	^a^	−3.67
Autophagy-related protein 2 homolog B	6.46	6.31	5.79	3.82	4.29	3.75
Lysosome membrane protein 2-like	4.27	4.72	3.11	4.39	4.03	3.55
Lysosomal alpha-mannosidase-like	2.34	2.56	2.51	9.49	10.11	10.05
Lysosomal-trafficking regulator-like	1.92	1.51	1.43	4.85	4.68	4.7
ADP-ribosylation factor-like 1	1.72	1.42	1.58	6.12	6.09	6.43
ADP-ribosylation factor-like 3	2.32	1.51	2.68	4.25	4.61	4.52
Beta-galactosidase-like	1.66	1.42	1.78	4.11	4.01	3.61

^a^means no significantly different in gene expression. The numbers in the Table represent Log_2_ fold change of I-SUS-BW/H-BW, II-SUS-BW/H-BW and III-SUS-BW/H-BW

### Expression validation using qRT-PCR

**Fig. 4:**
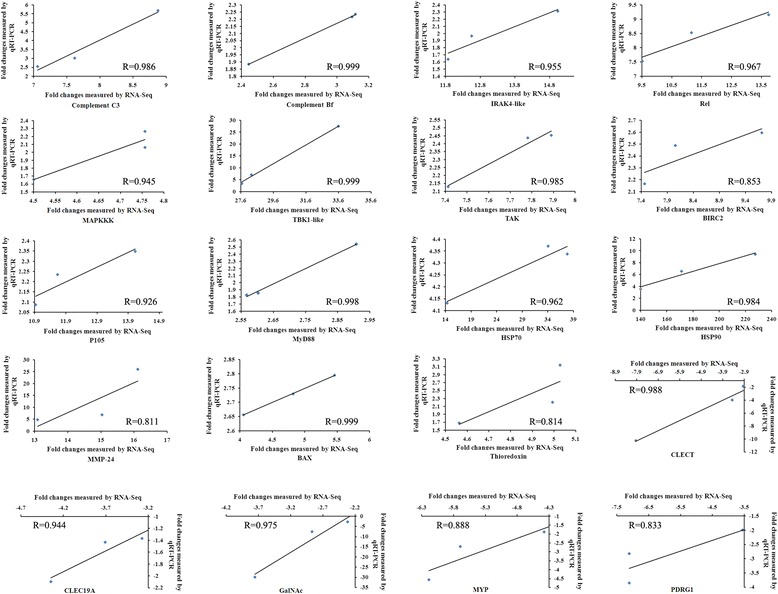
Validation of RNA-seq results using qRT-PCR. Twenty DEGs were selected and their relative fold changes were expressed as the ratio of gene expression in BW of *A. japonicus* at SUS stages I, II and III compared to H samples as normalized with the Cytb gene. The data obtained from the first RNA-seq and qRT-PCR were compared correspondingly and drawn as a Pearson correlation scatter plot

To validate the reliability of the RNA-seq results, twenty DEGs from Table 4 were detected by qRT-PCR at three stages of SUS progression (SUS stage I, II and III) and H samples. The cytochrome b (Cyt b) gene was chosen as the reference gene [32]. The 20 DEGs were selected for their clear background information in the function of immune responses, and some of them were involved in the complement pathway, TLR signaling pathway and Apoptosis. The Pearson's correlation coefficient (R) was used to assess the consistency of DEGs expression profiles between these two methods. All the DEGs at three stages of SUS progression showed a consistent expression pattern with R value ranging from 0.811 to 0.999 (Fig. 4).

## Discussion

### Changes in gene expression profiles during SUS progression

Using RNA-seq we have generated an extensive transcriptome profile for SUS-affected *A. japonicus*. The transcriptome profile allowed us to look at the changes in gene expression associated with the progression of the disease. Through construction of the first 13 cDNA libraries and comparison of the DEGs in four different tissues taken from SUS-affected *A. japonicus* against those of healthy *A. japonicus* samples, as well as investigation of the transcriptome associated with the three disease stages, we acquired a broad understanding of the dynamic transcriptome during the progression of SUS. Recently, 4,858 DEGs were found in *A. japonicus* coelomocytes by comparisons of SUS diseased and healthy control sea cucumbers. These genes were significantly enriched in tyrosine transport (biological progression), melanosome membrane (cell component), and L-tyrosine transmembrane transporter activity and 1-3-beta-glycan binding (molecular function) [28]. Gao et al. identified 102 DEGs in *A. japonicus* coelomocytes of SUS-affected group by comparing with that in control group. Many DEGs were involved in immune related pathways, such as Endocytosis, Lysosome, MAPK, Chemokine and the ERBB signaling pathway [29]. However, our data showed that between the SUS and healthy individuals, the biggest difference in expression profiles was found in BW. White skin ulceration is the main symptom of SUS; therefore, we further analyzed the changes in the transcriptome in the BW of sea cucumber during SUS progression from stage I to stage III.

### Pathways and DEGs during SUS progression

During the progression of SUS caused by *V. splendidus* challenge*,* immune regulation was an important event in this host-pathogen interaction process. In fact, most of the significantly enriched pathways involved in the immune system were observed in the up-regulated expression model during SUS progression. Of the eight significantly enriched pathways that were related to immune system, the “TLR pathway” has been reported in *A. japonicus* [32–35] response to bacterial challenge and in sea star *Pycnopodia helianthoides* response to sea star wasting disease [36]. Several signal transduction pathways in the up-regulated expression model during SUS progression, including the insulin [37], TGF-beta [38], MAPK [29], NF-kappa B [39, 40], and Notch [41] signaling pathways, have been reported to involve in innate immunity in invertebrate. In the studies, we further investigated DEGs involved in attachment/pathogen recognition, inflammatory reactions and apoptosis.

#### Attachment/pathogen recognition

Immune responses against invasive pathogens depend primarily on the recognition of pathogen components by innate pattern recognition receptors (PRRs). C-type lectins, which are a type of PRRs involved in innate immunity, specifically recognize sugars on the surface of pathogens in the presence of Ca^2+^, and cause a series of immune responses to resist pathogen invasion [42]. It is worth noting that four C-type lectins, including GalNAc-specific lectin [43], showed down-regulated expression and presented a downward trend during SUS progression (Table 4). Mannan-binding C-type lectin expression was also down-regulated at 48 h and 72 h after LPS challenge in *A. japonicus* [32]. Further studies are required to clarify the correlation between negative-regulation of this group of receptors and the occurrence of SUS in *A. japonicus*.

Cell adhesion molecules play important roles in many facets of the immune system. Integrins are the largest family of cell adhesion molecules that mediate cell-to-cell and cell-to-matrix interactions in a broad range of physiological and pathological processes [44]. The essential roles of integrins in developmental and innate immunity in invertebrates have been well studied, especially in phagocytosis [45, 46], encapsulation [47] and degranulation [48]. Integrin β1 is upregulated in hemocytes in response to various microbes in *Spodoptera exigua* [49] and integrin β is also involved as a cell adhesion receptor in the immune responses against microbe challenge in the shrimp *Litopenaeus vannamei* [50, 51]. Intriguingly, six integrin family genes were up-regulated during SUS progression in *A. japonicus*. The functions of integrins in *A. japonicus* response to SUS need to be further studied.

#### Inflammatory reactions

The innate immune system is the host’s first line of defense against infection. It triggers diverse humoral and cellular activities via signal transduction pathways which are conserved both in invertebrates and vertebrates [52]. In our studies, the DEGs included in inflammatory reactions were mainly involved in the complement pathway and TLR signaling pathway. The complement system is a major component of the innate immune system involved in defending against all the foreign pathogens [53]. The key genes of the complement system participating in this defense include complement components C3, C3-2, and factor B, all of which were up-regulated during SUS progression. In our earlier study, the expression of two complement component C3 genes in *A. japonicus* coelomocytes was also clearly up-regulated after the animal was challenged with LPS [54]. Zhong et al. reported that LPS challenge of *A. japonicus* induced significant up-regulation of the expression of a homologue of complement B factor (*AjBf-2*) in the coelomocyte and body wall [55]. C3 plays a pivotal role in the activation of both the classical and alternative complement pathways as well as the lectin pathways [56]. As the second complement component in the alternative pathway, Bf binds to the activated C3b and C3(H_2_O) [57]. In the sea urchin, these two complement components may be part of a simple complement system that is homologous to the alternative pathway in vertebrates [58].

The essential role of TLRs in the activation of innate immunity by recognizing specific patterns of microbial components is well established [59]. While individual TLR genes were not captured, downstream signaling components traditionally associated with TLR pathway were found. For up-regulated DEGs, the important molecules of this pathway, such as MyD88, IRAK4-like, TOLLIP, TAK, NF-kB p105 subunit, NF-kB Rel and stress-activated protein kinase JNK-like, were screened during SUS progression. It is noteworthy that TRAF1 and TRAF5-like, and serine/threonine-protein kinase TBK1-like were associated with the MyD88-independent TLR pathway. Two adaptor molecules in the *A. japonicus* Toll signaling cascade, MyD88 and TRAF6, have been isolated and characterized. The expression levels of these two genes have been shown to increase significantly during *V. splendidus* challenge [33]. The NF-kB homologues, *Aj-rel* and *Aj-p105,* have also been identified, and shown to be involved in LPS-induced immunity in *A. japonicus* [34]. The important molecules in this pathway such as MyD88, NF-kB Rel, TRAF6 and LPS-induced TNFα were all shown to be differentially expressed in *A. japonicus* coelomocytes after LPS challenge by RNA-seq analysis in our early studies [32]. As to the MyD88-independent pathway, two Toll-like receptor genes (*AjTLR3* and *AjToll*) were identified, which were functionally involved in the immune responses against Gram-positive and Gram-negative bacteria, fungi and double-stranded RNA (dsRNA) viruses [35].

#### Apoptosis

Apoptosis is an essential process in metazoans and is critical for the formation and function of tissues and organs [60]. Dysregulation of the apoptotic processes often leads to serious consequences in humans, such as neurodegenerative diseases [61], cancer [62], and autoimmunity [63]. Some apoptosis-related proteins, such as sarcoma oncogene (Src), vitronectin and vinculin, have been identified and displayed time-dependent depressed expression in the coelomocytes of *V. splendidus*-challenged *A. japonicus* [28]. In the present study, we identified the DEGs involved in the apoptosis pathway and determined their expression profiles during SUS progression. There are two major pathways leading to apoptosis: an extrinsic pathway initiated by death receptors and an intrinsic pathway that occurs through the mitochondria. In the extrinsic pathway, procaspase 8 is activated by receptors for FasL and TNF through the recruitment of intracellular death domain-containing proteins, such as FADD. In the intrinsic pathway, procaspase 9 is activated by cytochrome C released from the damaged mitochondria [64]. Our results showed down-regulated cytochrome C expression during SUS progression. Apoptosis regulator Bcl-2 is a family of evolutionarily related proteins that govern mitochondrial outer membrane permeabilization and perform either pro-apoptotic (such as BAX) or anti-apoptotic (such as Bcl-2) functions. Interestingly, our studies showed that these two opposing regulators were up-regulated expression during SUS progression. Another important apoptosis regulator, baculoviral inhibitor of apoptosis repeat-containing protein 2 (BIRC2), was identified with up-regulated expression during SUS progression. In addition, expressions of two transcription factors NF-kB p105 subunit and NF-kB Rel that suppress apoptosis were also up-regulated during SUS progression. In the study of human cancer, molecular targeting therapies have been focused on the regulation of apoptosis by Bcl-2 family proteins [65], IAPs [66] and NF-kB [67]. Since apoptotic signals are complicated and regulated at several levels, the mechanism underlying the regulation of apoptosis in SUS of sea cucumber is worthy of further exploration.

## Conclusion

The development of SUS in sea cucumber is a complex process in which tens of thousands of genes showed significantly different expression during the progression of the disease. Systematic investigation of the polygenic regulation and multiple pathways involved in the multi-stage progression of SUS is required to elucidate the dynamic mechanism of SUS progression. The findings reported in this study will be useful for further studies into the origins and development of SUS in sea cucumber. In further investigations, we will focus on the network biology approach to comprehensively depict the miRNA-mRNA and mRNA-protein networks relevant to SUS in *A. japonicus*.

## Methods

### Animals

Animals used in this research were obtained from commercial sea cucumber catches, therefore approval from any ethics committee or institutional review board was not necessary. Healthy *A. japonicus* (10–12 g) were collected from Zhuanghe, Liaoning Province (China). The animals were acclimated in the laboratory for 1 week, and then subjected to artificial infection. The sea water used in the experiment was filtered through sand, and then through 300-μm nylon sieves. Twenty-five percent of the seawater in the tank was exchanged daily. The animals were maintained at 12 °C, pH 8.1, with salinity of 32 and continuous aeration.

### Artificial infection and sample collection

*V. splendidus* used to infect *A. japonicus* was previously isolated and identified in our laboratory from SUS-affected *A. japonicus* according to the method reported by Deng et al. [22]. The bacteria were cultivated in 2216E medium at 28 °C for 24 h. The bacterial cells were then harvested by centrifugation at 1,000 × *g* for 5 min, and then re-suspended in 0.22-μm-filtered seawater. For the wounded immersion infection, 200 healthy *A. japonicus* individuals were cultured in five rectangular tanks (80 cm in length, 45 cm in width, and 45 cm in height). A cut (0.5 cm × 0.5 cm) was made in the body wall of the sea cucumbers using a scalpel and *V. splendidus* was added to the tank (final concentration, 5 × 10^9^ CFU mL^−1^), with 25 % seawater replenished daily; *V. splendidus* was supplemented and the bacterial concentrations were maintained. In the sample collection, white skin ulceration was considered to be the most important mark to distinguish diseased and healthy individuals [15, 28]. The progression of SUS was divided into three stages. In stage I, the animals showed one small white speck of skin ulceration (diameter <0.2 cm). The animals retained the ability to attach to the surface of the tank and did not eviscerate. In stage II, the animals exhibited 2 to 3 larger white specks (diameter >0.2 cm). The animals continued to exhibit the ability to attach to the surface of the tank and did not eviscerate. In stage III, the individuals showed several deep and extensive ulcerations, lost the ability to adhere to the tank and displayed evisceration. *A. japonicus* that cultured under normal conditions without the treatments of cut and bacterial challenge served as healthy controls. Thirty individuals were selected from each of the SUS stages (stage I, stage II, and stage III) and the ulcerative body wall and its distal normal tissue were sampled for RNA analysis (Fig. 1). In addition, samples of the Int, RT and C were also obtained at SUS stage II. The C were collected by centrifugation at 1,000 × *g* for 5 min. Samples of BW, Int, RT and C were separated from 30 healthy *A. japonicus* individuals. For each sampled individual, the size of BW tissues is about 0.2 cm × 0.2 cm × 0.2 cm. The quality of sampled Int and RT tissues is less than 100 ng. The volume of C is about 1 mL. All samples were frozen immediately in liquid nitrogen and then stored at −80 °C before RNA isolation.

### RNA extraction, cDNA library construction and sequencing

Total RNA was extracted from the specimens with Trizol reagent (Invitrogen, Carlsbad,CA,USA) according to the manufacturer’s instructions. The quality and concentration of total RNA were measured by Nanodrop 1000 (Thermo). The initial amount of high-quality total RNA was 1 μg per library (generated from 30 individuals)*.* Subsequently, the mRNA in the total RNA was enriched using Oligo (dT) magnetic beads and sheared into short fragments by adding fragmentation buffer, followed by first- and second-strand cDNA synthesis using random hexamer primers. The cDNA fragments were subjected to an end-repair process, addition of “A” base*,* and ligation of sequencing adapters. After agarose gel electrophoresis, suitable fragments were selected and used as templates for the PCR amplification to create the final cDNA libraries. We first constructed 13 cDNA libraries (from D1 to D13), representing the ulcerative BW and their distal normal tissues at all three stages of SUS development (Fig. 1) and four tissues (BW, Int, RT and C) of SUS-affected and healthy samples. High-throughput sequencing was conducted using the Illumina HiSeq™ 2000 platform to generate 50-bp reads.

### Data processing, assembly and annotation

The original image data were converted to sequence data by base-calling and saved as fastq files. The raw reads were then cleaned by discarding adaptors, low-quality reads (quality scores <20), reads with unknown bases greater than 5 %, and reads of less than 20 nt. De novo transcriptome assembly combined with Illumina reads obtained from GenBank [28, 30] was carried out with the short-read assembly program, Trinity [68]. The assembled sequences from the Illumina reads, 454 sequences and ESTs were used in further processing of the assembly according to the method reported by Zhou et al. [30]. Blastx analysis of unigenes longer than 200 bp was conducted against the Non-Redundant (Nr) database, Swiss-Prot, COG and KEGG database with an *e*-value cutoff of 1E-5. GO classification was analyzed by Blast2GO software (v.2.5.0) based on best hits of Nr annotation. In addition, transcripts were also annotated with the NCBI non-redundant nucleotide (Nt) database using Blastn with an *e*-value cutoff of 1E-5.

### Screening and analysis of DEGs

The unigene expression was calculated using the RPKM (Reads Per kb per million reads) method [9]. To identify the DEGs in the first 13 *A. japonicus* libraries, a rigorous algorithm was developed for statistical analysis according to “the significance of digital gene expression profiles” [69]. The FDR (false discovery rate) is used in multiple hypothesis testing to correct for *P*-values [70]. In our analysis, the FDR ≤ 0.001 and RPKM ratio larger than 2 (|log_2_Ratio| ≥ 1) were used as the threshold to judge the significance of differences in gene expression. The dynamic expression profiles of DEGs during the three-stage SUS progression were visualized by clustering analysis, based on the K-means method using the Euclidean distance algorithm. The enrichment analysis was carried out based on an algorithm presented by KOBAS [71], with the entire transcriptome set as the background. The *P*-value was approximated by a hypergeometric distribution test and FDR multipletesting correctionwas used to corrected-*P*-value [70]. The GO enriched cutoff was a corrected-*P*-value ≤ 0.05, and the KEGG enriched cutoff was a Q value ≤ 0.05.

### Transcriptome sample preparation, re-sequencing and DEG identification

After analysis of the gene expression profiles in the first 13 cDNA libraries, we re-sequenced samples of the ulcerative BW of the three SUS stages and healthy BW. The three stages of SUS-affected samples were also infected with *V. splendidus*. Subsequently, ulcerative BW of individuals during the three SUS stages and healthy BW were separated for cDNA library construction (from T1 to T8) and RNA-seq. Two biological replicates of each sample were set up. High-throughput sequencing was conducted using the Illumina HiSeq™ 2000 platform to generate 100-bp paired-end reads. After quality control for raw reads, clean reads were mapped to the reference transcriptome and their abundance estimated using RSEM (RNA-seq by expectation maximization). DEGs were detected by using the DESEQ program from the R-Bioconductor package.

### Expression validation using qRT-PCR

To validate the RNA-seq results, 20 DEGs was employed for qRT-PCR (Mx3005p™ detection system, Agilent Stratagene, Santa Clara, CA, USA). Total RNA from samples used in the first RNA-seq was reverse-transcribed into cDNA templates with the PrimeScript™ RT reagent Kit (TaKaRa, Otsu, Japan) according to the manufacturer’s instruction. Reactions were incubated at 37 °C for 15 min, and then 85 °C for 5 s. According to the sequence information in the transcriptome database, primers were designed using Primer 5.0 software according to rigorous criteria. The primer information was provided in Table 5. The cytochrome b (Cytb) gene was chosen as the reference gene [32]. Optimal primer pairs were examined by checking the melting curve at the end of each PCR reaction to confirm the specificity of PCR product. The qRT-PCR amplification was conducted in a volume of 20 μL containing 10 μL of 2× SYBR Premix Ex Taq™ II (*Tli* RNaseH Plus, TaKaRa, Otsu, Japan), 0.4 μL of ROX Reference Dye II, 2 μL of cDNA template, and 0.4 μM of each primer according to the introduction. Thermal cycling was as follows: 95 °C for 30 s, 40 cycles at 95 °C for 10 s, 56 °C for 25 s and 72 °C for 25 s. All reactions were run in triplicates. Relative Expression Software Tool 384 v.2 (REST) (Technical University of Munich, Munich, Germany) [72] was used to calculate the expression differences between control and SUS groups (SUS stage I, II and III). The correlation of DEGs expression profiles between RNA-seq and qRT-PCR was assessed by Pearson correlation coefficient (R). The nearer the scatter of points is to the straight line, the higher the strength of association between the variables. The value of R ranges from −1 to +1 [73, 74].

**Table 5:** Primers used for qRT-PCR validation

Gene	Primer Sequence (5′-3′)
Cytochrome b	Cytb-F: TGAGCCGCAACAGTAATC
	Cytb-R: AAGGGAAAAGGAAGTGAAAG
Complement component 3	AjC3-F: GCGTTGTTTCGTTCAACAAGGGGA
	AjC3-R: GCCATTCACTGGAGGTGTGCCA
Complement factor B	Bf-F: ATTATCTCGCAACAGCGATCC
	Bf-R: GGGCAACCACACCGGCTTCTCCA
IRAK4-like	IRAK4-F: TACACGTCAGATCGGGATGA
	IRAK4-R: TAAACGACGAGCGTACCACA
NF-kB transcription factor Rel	Rel-F: TGCGAAGCCACATCCATT
	Rel-R: AGGGCATCCTTTAAGTCAGC
MAPKKK	MAPKKK -F: GAATCAGAGGAGATAGATGTGGAGA
	MAPKKK -R: AGGAGGAGGAGGAAGACGAC
Serine/threonine-protein kinase TBK1-like	TBK1-F: AGATGATGTTGTCCATTCTCG
	TBK1-R: ACAGGAGGAAGTGATGTGCT
TGF beta-activated kinase	TAK -F: TCTCTGTAGCCTCCTTTGACG
	TAK -R: CTCGGTCTTCCAACCAACAC
BIRC2	BIRC2-F: TCAGGCACGAGTGACAAAGT
	BIRC2-R: GCATGAGCCATTCACATCTCA
NF-kB p105 subunit	p105-F: GCAACACACCCCTCCATCTT
	p105-R: TCTTCTTCGCTAACGTCACACC
MyD88	MyD88-F: CCGATGTAGGAGGATGGTAGTAG
	MyD88-R: CACAGTAAGGTGCTGAAGAATGC
HSP 70	HSP 70-F: AAGAGCACAGGCAAAGAG
	HSP 70-R: TGATGATGGGTTGGCACA
HSP 90	HSP 90-F: TATGAAAGCCTGACAGACGCAAGC
	HSP 90-R: TAACGCAGAGTAAAAGCCAACACC
Matrix metalloproteinase-24	MMP-F: CGATTCAGTCTTCCCTGGTG
	MMP-R: ACCGTCATCAACTTTCCTGGT
Apoptosis regulator BAX-like	BAX-F: GCCGTGGGACTGACTTTACA
	BAX-R: TCCATCTCGTAGTTCTCTCAACG
Thioredoxin	TRx-F: GCTGGTGACAAACTGGTGAT
	TRx -R: TGAGAAAGACAACGTCGGTA
CLECT	CLECT-F: GACGGCTTGTCCAGAGTT
	CLECT-R: AGGTCCATTGTTGGGTTC
CLEC19A	CLEC19A-F: ATGCAGCGAGAAGATGGAGT
	CLEC19A-R: TGGCAGGATATGCCCTAGAT
GalNAc-specific lectin	GalNAc -F: CCATCCTTCAGGGCAGATAA
	GalNAc -R: TTCATCGACCAAAATGCAGA
Major yolk protein	MYP-F: AGGAGGGAGACATTGCTT
	MYP-R: ATGATGCTTTCTGGGTTG
PDRG 1	PDRG1-F: AATTGGAGGAACTCGCTGAA
	PDRG1-R: TTGCTTATCGCCTTCTTGTG

## Abbreviations

BAX, apoptosis regulator BAX-like; Bf, complement factor B; BFAR, Bifunctional apoptosis regulator; BIRC, baculoviral IAP repeat-containing protein; BW, body walls; C, coelomocytes; C3, complement component C3; C3-2, complement component 3–2; CLEC19A, c-type lectin domain family 19 member A; CLECT, c-type lectin; DEGs, differentially expressed genes; DRAM, DNA damage-regulated autophagy modulator protein; FNDC3A, fibronectin type-III domain-containing protein 3A; GalNAc, alpha-N-acetylgalactosamine-specific lectin; GO, gene ontology; GPX, glutathione peroxidase; HSP, heat shock protein; IAP, inhibitor of apoptosis; IFI27, interferon alpha-inducible protein 27; IGDCC4, immunoglobulin superfamily DCC subclass member 4; Int, intestines; IRAK4, interleukin-1 receptor-associated kinase 4; KEGG, kyoto encyclopedia of genes and genomes; LENG, leukocyte receptor cluster member8; LIG-3, leucine-rich repeats and immunoglobulin-like domains protein 3; MAPKKK, mitogen-activated protein kinase kinase kinase; MMP, matrix metalloproteinase; MyD88, myeloid differentiation factor 88; MYP, major yolk protein; NFIL3, nuclear factor interleukin-3-regulated protein; P105, NF-kB p105 subunit; PDCD, programmed cell death protein; PDCD6IP, programmed cell death 6-interacting protein; PDRG1, p53 and DNA damage-regulated protein 1; PRR, pattern recognition receptor; Rel, NF-kB transcription factor Rel; RNA-seq, RNA-sequencing; RT, respiratory trees; SAPK, stress-activated protein kinase; SRCR, scavenger receptor cysteine-rich; SUS, skin ulceration syndrome; TAK, TGF-beta-activated kinase; TBK1, serine/threonine-protein kinase TBK1; TLR, toll-like receptor; TMPRSS, transmembrane protease serine; TOLLIP, toll-interacting protein; TRAF, TNF receptor-associated factor.

## Additional files

Additional file 1: Significantly enriched GO terms in DEGs shown by the up-regulated expression models (xls). (XLS 52 kb) Additional file 2: Significantly enriched pathways among the DEGs revealed by the up-regulated expression models (xls). (XLS 29 kb) Additional file 3: Toll-like receptor signaling pathway (tif). Red boxes represent up-regulated genes, and green boxes represent down-regulated genes. (TIF 482 kb) Additional file 4: Complement and coagulation cascades pathways (tif). Red boxes represent up-regulated genes, and green boxes represent down-regulated genes. (TIF 627 kb) Additional file 5: Apoptosis pathways (tif). Red boxes represent up-regulated genes, and green boxes represent down-regulated genes. (TIF 421 kb) Additional file 6: Apoptosis pathways (tif). Red boxes represent up-regulated genes, and green boxes represent down-regulated genes. (TIF 236 kb) Additional file 7: Pearson correlations between samples (tif). (TIF 417 kb) Additional file 8: Visual changes of global DEGs at three stages of SUS progression in *A. japonicus* (tif). Green points represent up-regulated genes, and red points represent down-regulated genes. (TIF 624 kb)
