# Supplementary figures and images for: RNA sequencing analysis to capture the transcriptome landscape during skin ulceration syndrome progression in sea cucumber Apostichopus japonicus
Source: BMC Genomics. 2016 Jun 14;17:459. doi: 10.1186/s12864-016-2810-3 (PMC4906609; doi:10.1186/s12864-016-2810-3)

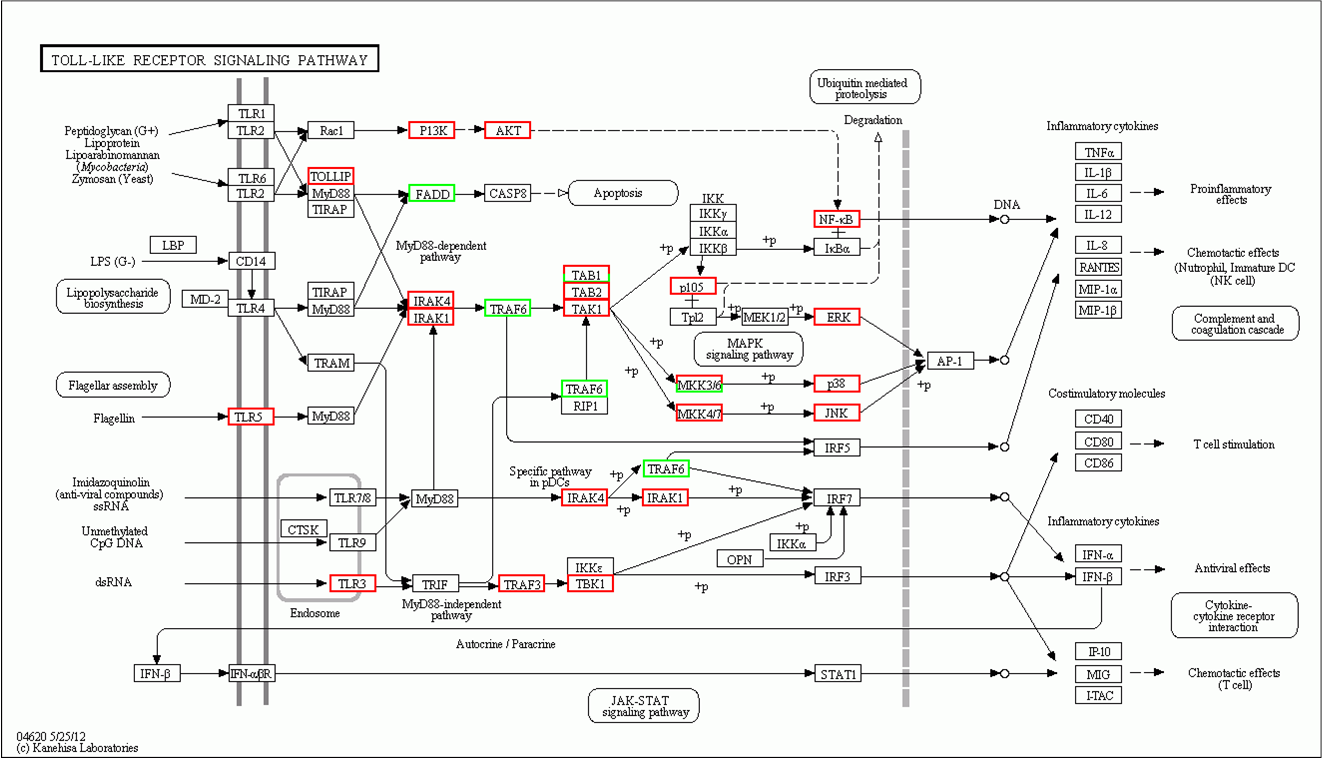

Supplement: Additional file 3: — Toll-like receptor signaling pathway (tif). Red boxes represent up-regulated genes, and green boxes represent down-regulated genes. (TIF 482 kb) [file 12864_2016_2810_MOESM3_ESM.tif]

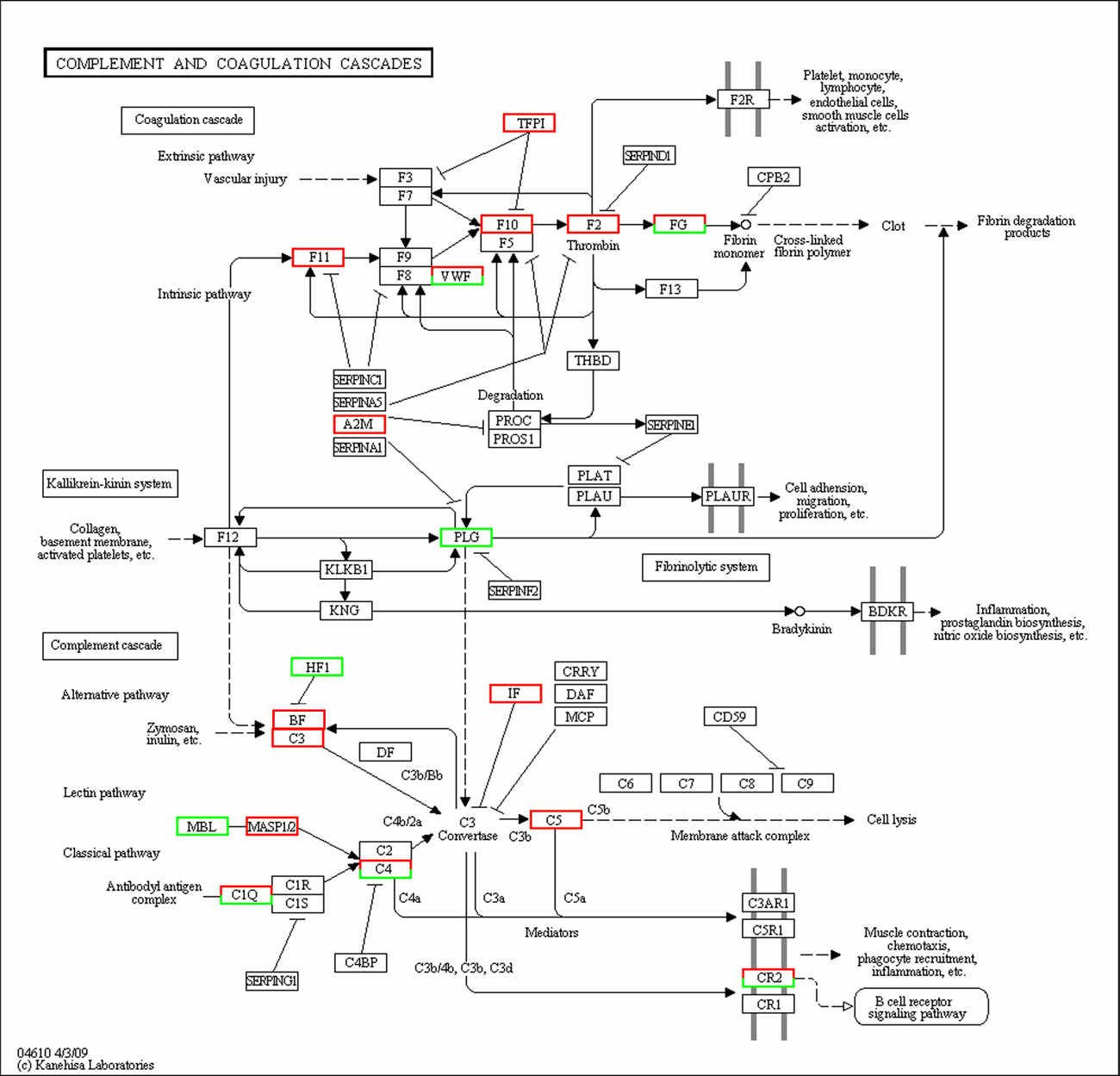

Supplement: Additional file 4: — Complement and coagulation cascades pathways (tif). Red boxes represent up-regulated genes, and green boxes represent down-regulated genes. (TIF 627 kb) [file 12864_2016_2810_MOESM4_ESM.tif]

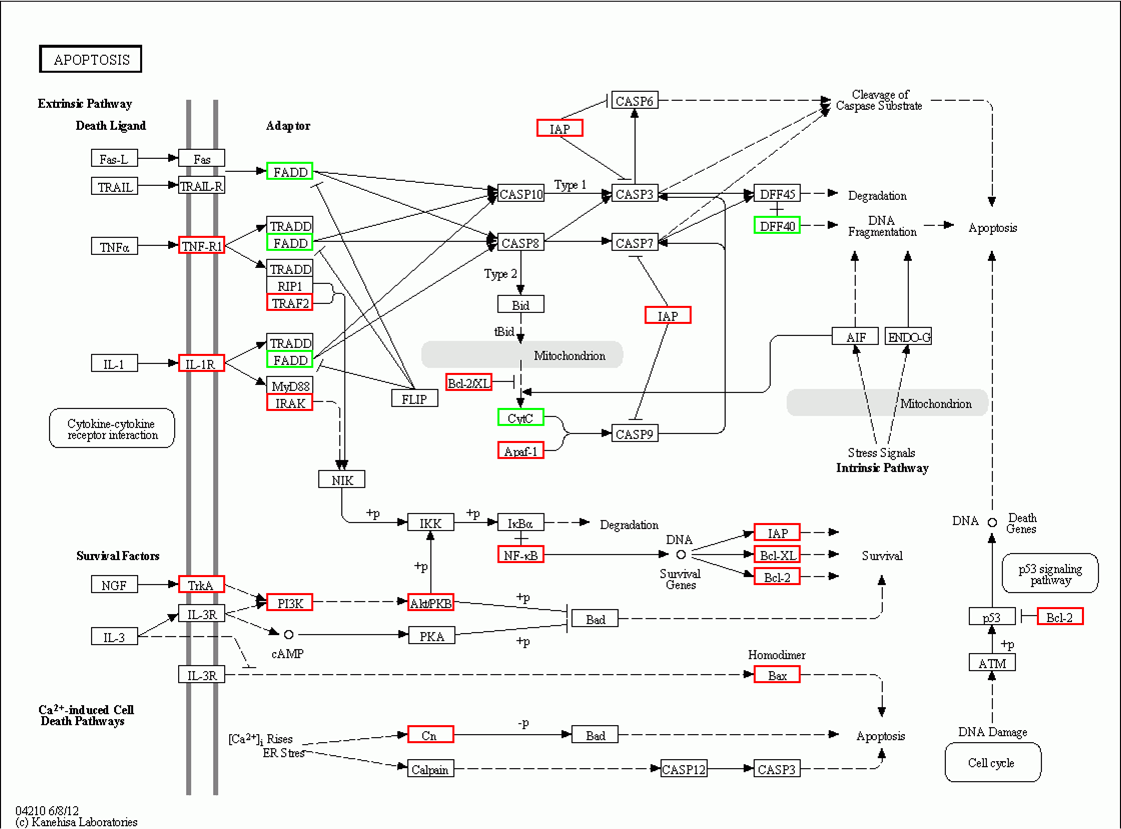

Supplement: Additional file 5: — Apoptosis pathways (tif). Red boxes represent up-regulated genes, and green boxes represent down-regulated genes. (TIF 421 kb) [file 12864_2016_2810_MOESM5_ESM.tif]

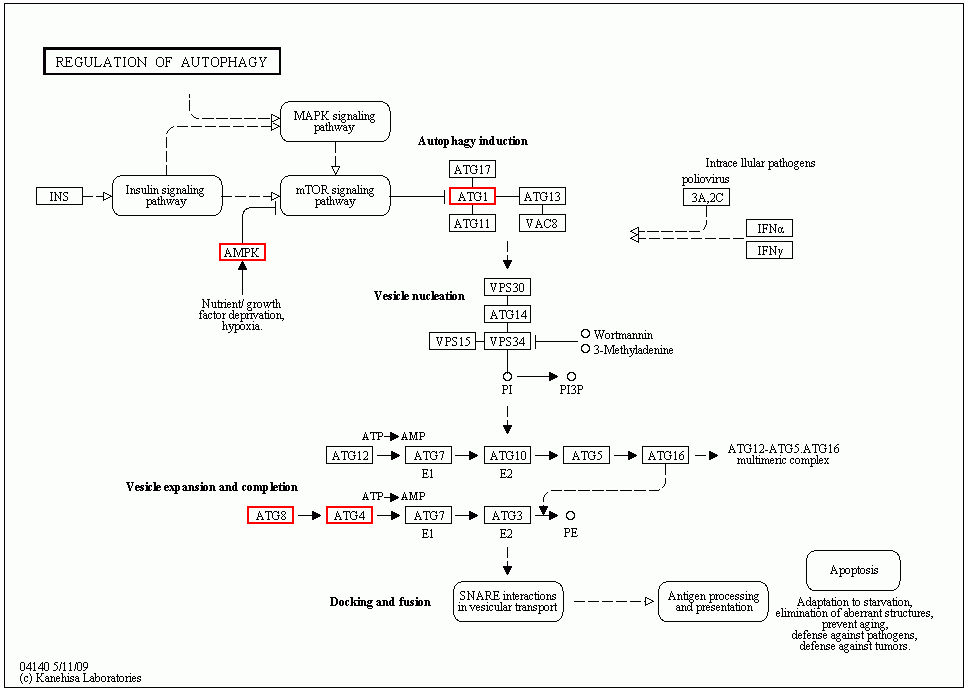

Supplement: Additional file 6: — Apoptosis pathways (tif). Red boxes represent up-regulated genes, and green boxes represent down-regulated genes. (TIF 236 kb) [file 12864_2016_2810_MOESM6_ESM.tif]

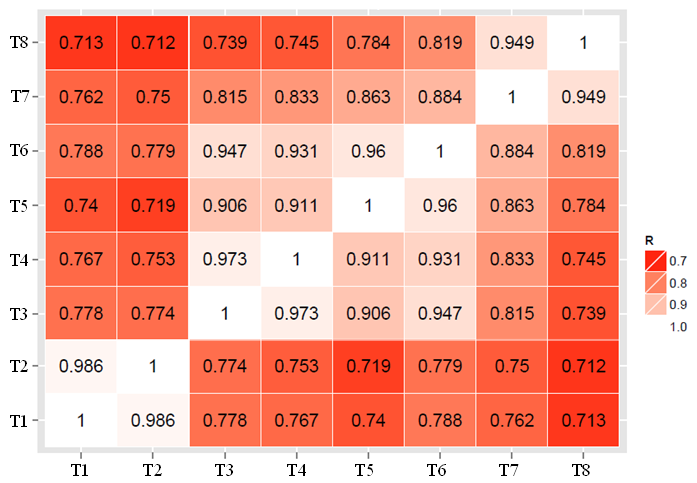

Supplement: Additional file 7: — Pearson correlations between samples (tif). (TIF 417 kb) [file 12864_2016_2810_MOESM7_ESM.tif]

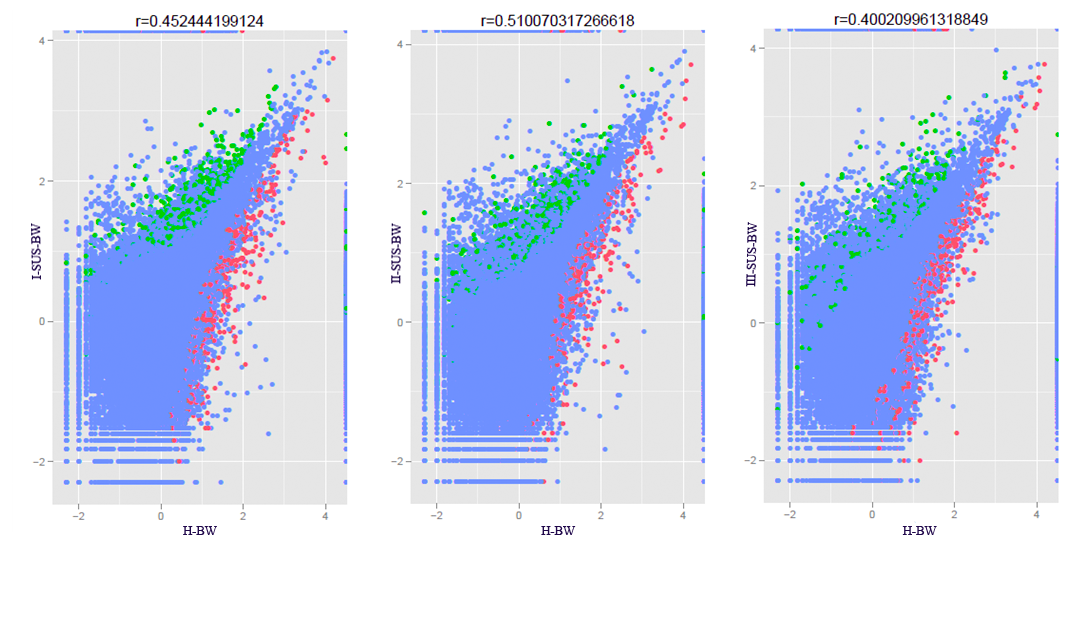

Supplement: Additional file 8: — Visual changes of global DEGs at three stages of SUS progression in A. japonicus (tif). Green points represent up-regulated genes, and red points represent down-regulated genes. (TIF 624 kb) [file 12864_2016_2810_MOESM8_ESM.tif]
